# Establishment of Humanized Mice for the Study of HBV

**DOI:** 10.3389/fimmu.2021.638447

**Published:** 2021-02-19

**Authors:** Fritz Lai, Cherry Yong Yi Wee, Qingfeng Chen

**Affiliations:** ^1^Institute of Molecular and Cell Biology, Agency for Science, Technology and Research (A*STAR), Singapore, Singapore; ^2^Department of Medicine, Yong Loo Lin School of Medicine, National University of Singapore, Singapore, Singapore; ^3^Key Laboratory for Major Obstetric Diseases of Guangdong Province, The Third Affiliated Hospital of Guangzhou Medical University, Guangzhou, China; ^4^Department of Physiology, Yong Loo Lin School of Medicine, National University of Singapore, Singapore, Singapore

**Keywords:** HBV, humanized mice, human immune system, human liver chimeras, chronic inflammation, liver fibrosis, HCC development, human hepatocyte

## Abstract

Viral hepatitis particularly Hepatitis B Virus (HBV) is still an ongoing health issue worldwide. Despite the vast technological advancements in research and development, only HBV vaccines, typically given during early years, are currently available as a preventive measure against acquiring the disease from a secondary source. In general, HBV can be cleared naturally by the human immune system if detected at low levels early. However, long term circulation of HBV in the peripheral blood may be detrimental to the human liver, specifically targeting human hepatocytes for cccDNA integration which inevitably supports HBV life cycle for the purpose of reinfection in healthy cells. Although there is some success in using nucleoside analogs or polyclonal antibodies targeting HBV surface antigens (HBsAg) in patients with acute or chronic HBV^+^ (CHB), majority of them would either respond only partially or succumb to the disease entirely unless they undergo liver transplants from a fully matched healthy donor and even so may not necessarily guarantee a 100% chance of survival. Indeed, *in vitro/ex vivo* cultures and various transgenic animal models have already provided us with a good understanding of HBV but they primarily lack human specificity or virus-host interactions in the presence of human immune surveillance. Therefore, the demand of utilizing humanized mice has increased over the last decade as a pre-clinical platform for investigating human-specific immune responses against HBV as well as identifying potential immunotherapeutic strategies in eradicating the virus. Basically, this review covers some of the recent developments and key advantages of humanized mouse models over other conventional transgenic mice platforms.

## Introduction

Hepatitis B Virus (HBV) infection remains a major health threat globally that contributes extensively to various types of liver diseases primarily due to development of acute hepatitis B which progresses into chronic hepatitis B (CHB) and subsequently causes liver fibrosis, cirrhosis and hepatocellular carcinoma (HCC). Based on the Global Hepatitis Report by WHO in 2015, it is estimated that 257 million people in the world are currently living with CHB (1). In fact, viral hepatitis was firmly responsible for 1.34 million deaths which was also the 6th leading cause of death worldwide overtaking the number of deaths caused by HIV and tuberculosis which was ranked at 7th and 12th, respectively (2). HBV can be classified into 10 Genotypes (A to J) depending on various geographical regions for example, Genotype A is highly endemic in areas like Africa, Europe, India, and America whereas Genotypes B and C are commonly found in the Asia-Pacific region (3). Genotype D is prevalent in Africa, Europe, the Mediterranean region, and India but Genotype E is only identified in West Africa. On the other hand, Genotype F is limited to Central and South America. In addition, there have been reports of Genotype G profiles in France, Germany, and the Americas however, Genotype H is mainly found in Mexico and Central America. Finally, Southeast Asian countries like Vietnam and Laos did portray patients with HBV Genotype I whereas Genotype J has only been reported in Japan. Although some countries are more susceptible to specific HBV genotypes, it is vital to note the mode of viral spread particularly in Asian regions. Genotypes B and C are found in most Asian countries where HBV primarily infects infants at a perinatal stage or through mother-to-infant transmission (1, 4). Therefore, preventive measures like Hepatitis B vaccinations have been enforced worldwide (mandatory in most countries) during the first 5 years of the child's birth. It is especially important to provide vaccinations within this timeframe as the risk of HBV progression from acute to CHB can be as high as 95% at a perinatal period and 20–30% if infection occurs between the age of 1–5 (4). In fact, Singapore was one of the first countries in the world to have implemented the national childhood HBV immunization programme to all newborns as early as 1st September 1987 (5). This initiative had proven effective as the national hepatitis B seroprevalence study have recorded a significant reduction in HBV infection rates as well as increasing HBV immunity primarily in adult Singaporeans (aged 18–29 years) between 1998 and 2010. Of these young adults that were positive for hepatitis B surface antigen (HBsAg), none were positive for hepatitis B e antigen (HBeAg) in 2010 compared to 20.8% in 1998. Similarly, detection of hepatitis B core antigen (HBcAg) has also decreased significantly from 22.1 to 4.4% whereas immunized antibodies produced against HBsAg (anti-HBs) increased from 27.9 to 43.3%.

Despite implementation of such immunization programmes, an active infection can still occur. There is currently no curative treatment for patients with CHB but several antiviral agents like interferon (IFN)-α or pegylated (PEG)-IFN-α and nucleoside analogs (NUC) have been approved for long-term suppression of HBV viremia (6). Although these antiviral agents can reduce circulating HBV to below threshold of detection limit in majority of patients, complete eradication of HBV life cycle remained a challenge as intrahepatic replication proved to be primarily responsible for viral persistence, rebound of viremia during treatment withdrawal and ultimately progression of liver pathogenesis. In addition to the life-long physical symptoms of people living with CHB, the importance of psychosocial burden such as anxiety, financial loss, social discrimination, and rejection should also be recognized equally (7).

Therefore, many have utilized cell culture-based assays and various animal models to better understand specific aspects of the disease including HBV life cycle, virus-host interactions, immune responses, potential therapeutic targets, and more. It is clear that HBV naturally targets human hepatocytes which can be easily obtained commercially or isolated from liver resection of patients with CHB (6, 8). However, limited accessibility due to financial constraints, poor quality of liver tissues, individual preparation variabilities, etc., proved to be the biggest drawbacks. Hence, human hepatoma cell lines like Huh7 and HepG2 were widely used as an alternative to primary human hepatocytes (PHH) cultures to mainly study post-transcriptional involvement of HBV life cycle following transfection of plasmids carrying specific regions of HBV DNA. These cell lines are not actually susceptible to HBV infection due to the lack of expression of HBV receptors until the discovery of a key integral transmembrane protein, sodium taurocholate co-transporting polypeptide (NTCP) where viruses are known to enter human liver cells through binding of NTCP receptors at the basolateral membrane of hepatocytes prior to being engulfed (9, 10). This was further validated in NTCP-overexpressing cell lines that are more permissive to HBV infection which can be attenuated by gene silencing of NTCP or utilizing peptides like Mycludex B (MyrB) to block viral entry (10). In fact, NTCP was simply first identified through a basic understanding of the HBV genome being an enveloped virus encoding HBeAg, HBcAg, HBx, Pol, large surface (L), middle surface (M), and small surface (S) envelop proteins where the pre-S1 domain of the L protein was later recognized as a key determinant factor that promotes viral entry (9). Moreover, the full life cycle of HBV accomplished through generation of pregenomic RNA (pgRNA) during reverse transcription at overlength genome regions containing HBV core promoter is required for production of mature HBV viral particles (11). More importantly, the virological key to this endless infectious property of HBV is due to an intracellular HBV replication intermediate called covalently closed circular DNA (cccDNA) which is primarily responsible for viral persistence and reactivation following therapeutic withdrawal (12–14). Although the molecular mechanisms of cccDNA formation remains unclear, its fundamental characteristic in synthesizing new virions has already been described in cell culture settings as an episomal plasmid-like molecule that resides in host cell nucleus making it a prime target for elimination.

Indeed, both PHH and hepatoma cell lines have offered valuable insights on the study of HBV infection however, it is equally important to validate these findings *in vivo*. As we all know, HBV has a very limited host spectrum which makes investigation of viral tropism in animals very challenging. Only a handful of animals such as chimpanzee, Mauritian cynomolgus monkey, treeshrew, and woodchuck models have successfully harbor HBV infection to not only recapitulate liver disease pathogenesis (fibrosis, cirrhosis, and HCC) seen in HBV^+^ patients but also offers a more accurate representation of viral-host interaction (15–19). However, handling of these larger animals requires strict ethics approval and involves high maintenance cost hence, most labs prefer using small animal models like mice as they reproduce in large numbers and can be easily purchased commercially. The evolution of various mouse strains over the last 20 years has clearly emphasized the importance of having a reliable *in vivo* platform particularly evidenced by the constant improvement in generating a mouse model that encompasses human liver chimeras and/or human immune system to not only allow better identification of novel therapeutic strategies to combat HBV but also to elucidate mechanisms of human-specific viral-host immune responses. In this review, we will highlight some of the key humanized mouse model systems that have significantly enhanced the understanding of HBV research below.

## The Evolution of Humanized Mice

Prior to mouse humanization, HBV transgenic mice were frequently utilized to mainly evaluate various methods of HBV clearance through molecular manipulation of specific regions of HBV using siRNA/shRNA (20, 21). However, the major drawbacks of utilizing this system is the absence of HBV cccDNA in mouse hepatocytes and failure of mice to exhibit HBV-induced liver pathogenesis. Subsequently, HBV DNA delivery by hydrodynamic injection (plasmid DNA) and viral vectors like Adenovirus, Baculovirus, Adeno-associated viruses (AAV) improved HBV transfection efficiency, stability as well as maintenance for longer periods (22–25). Unfortunately, HBV cccDNA was again not detected in mouse hepatocytes suggesting that there could be some forms of impairment in the HBV cccDNA intracellular recycling pathway. It was only until recently when recombinant HBV cccDNA was successfully generated in mice injected hydrodynamically through Cre-/Loxp-mediated recombination which functions similarly to real HBV cccDNA in the production of mature viruses (14, 25). More importantly, the improved stability and persistence of HBV severely damaged mice livers for the very first time which resulted in advanced liver pathogenesis evidenced by development of fibrosis (26). Although transgenic mice exhibiting such phenotype was considered a major breakthrough in the field of HBV *in vivo*, the study still revolved around a complete mouse host setting. Since human hepatocytes are the natural cellular target of HBV, the inevitable transition of mouse to human eventually gave rise to various establishments of human liver chimeric mice.

### HBV-Trimera Mice

Mice engrafted with primary human cells have long been utilized for research such as xenograft transplants of human cancer cell lines into nude mice for the study of tumorigenesis *in vivo* (27). Over time, it was demonstrated that immunodeficient mouse recipients that lack mouse T, B and Natural Killer (NK) cells combined with an additional deletion of the common γ-chain of the interleukin 2 receptor (IL-2rγ) offered the most successful human engraftments with very low risk of rejection (28–30). So, the “Trimera” mouse was one of the earliest model involving the use of human hepatocytes in HBV study where wild-type mice were lethally irradiated prior to immediate injection of radioprotective bone marrow cells from SCID mouse followed by transplantation of *ex vivo* HBV-infected human liver tissues under the kidney capsule 10 days later (31). Although low levels of viremia were detected in these mice which can be reduced by human polyclonal anti-HBs antibody, Hepatect and reverse-transcriptase inhibitors, human hepatocytes only remained functional for a very short timeframe and that HBV persistence could not be fully established *in vivo*. Therefore, generation of a chimeric mouse liver model with robust expansion of human hepatocytes within the liver parenchyma would be key in permitting a stable HBV infection *in vivo*.

### uPA-SCID Transgenic Mice

By taking advantage of the liver's regenerative property, Albumin-urokinase-type plasminogen activator (uPA) transgenic mouse was the first model to successfully demonstrate repopulation of adult human hepatocytes transplanted from a healthy donor into the liver of a diseased mouse recipient (32, 33). Basically, this system of hepatocytes renewal relied on the creative concept of deliberately inducing hepatic injury in particular, to mouse hepatocytes in order to make space for healthy ones to accommodate the damaged liver. It was reported that the constitutive expression of murine uPA gene driven by an albumin enhancer/promoter was responsible for hepatotoxicity, elevated plasma uPA levels, hypofibrinogenemia, spontaneous intestinal and intra-abdominal hemorrhaging in neonates which was eventually utilized to facilitate mouse liver damage (34). In addition to overexpressing uPA transgene, these mice were backcrossed with an immunodeficient strain such as the Severe Combined Immune Deficient (SCID) that lacks functional B, T and NK cells to better permit reconstitution of xenogenic human hepatocytes in the liver (33, 35, 36). These mice were also capable of harboring high levels of HBV replication which later became an *in vivo* forefront of hepatitis research particularly in pre-clinical assessments of novel antiviral therapeutics (37–39). Although many had utilized various mouse strains in generating a similar uPA mouse model, the uncontrolled constitutive expression of this toxic gene resulted in poor breeding efficiency, limited timeframe for transplantation and high mortality whenever the transplanted human hepatocytes were unable to compensate for mouse hepatocyte cell death (40–42). Hence, the unpredictability in mice maintenance and high cost demands somewhat restricted wide application of this model.

### FRG Knockout Mice

Stability of the human liver chimeric mice system was gradually finetuned with the generation of Fah knockout (KO) mice in 2007 (43). Fumarylacetoacetate hydrolase (Fah) is a mouse-specific enzyme required for liver metabolism which primarily plays an important role in the last steps of the tyrosine catabolism pathway. Mice deficient in the Fah gene redirected its metabolic pathway to accumulate toxic tyrosine metabolic intermediates which subsequently damaged mouse hepatocytes (44–46). To maintain normal liver function, mice drinking water were supplemented with 2-(2-nitro-4-fluoromethylbenzoyl)-1,3-cyclohexanedione (NTBC), a chemical that has been approved to treat hereditary tyrosinemia type 1 (46). More importantly, NTBC drug can actually be passed down to pups through the mother's milk which greatly reduced the rate of mortality in pups of Fah KO mice. Unlike the uPA-overexpressed transgenic mice, NTBC cycling withdrawal offers a much better system in controlling the severity of mouse liver damage depending on the proliferative capability of post-transplanted human hepatocytes which can also be measured via human albumin expression in the blood (6, 11, 29, 43). To avoid any immune rejection risk of human cells, Fah KO mice were crossed into a Rag2/IL-2rγ double knockout strain which has been demonstrated to reconstitute human hematopoietic cells efficiently (47, 48). The newly generated Fah/Rag2/IL-2rγ triple KO mouse termed as FRG KO, were not only able to expand human hepatocytes robustly but also sustained high production of HBV in the serum without displaying any cytopathic pathogenesis (43). In addition to NTBC withdrawal, these mice were also administered with a urokinase-expressing adenovirus (ad-uPA) prior to animal surgery to further induce cell-autonomous hepatotoxicity for an enhanced human hepatocyte engraftment (49). Therefore, FRG KO mice have proven to be one of the most sought-after *in vivo* platforms for studying mechanisms of HBV infection and identification of novel antiviral therapeutics. In fact, our group has also recently utilized FRG KO mice to further investigate the concerted actions of IFN-α and -γ signaling and identified IFN-α14 as a potent interferon subtype for suppressing HBV (50).

### TK-NOG Transgenic Mice

A similar drug-induced human liver chimeric mouse model was established in 2011 where the herpes simplex virus type 1 thymidine kinase (UL23 or HSVtk) gene driven by a mouse albumin promoter was specifically expressed in livers of severely immunodeficient NOG mice (TK-NOG) (51, 52). TK-NOG mice were briefly exposed to the non-toxic drug ganciclovir (GCV) to selectively destroy mouse hepatocyte cells that were expressing the HSVtk transgene thereby allowing space for the transplanted human hepatocytes to repopulate the liver. As HSVtk only catalyzes GCV phosphorylation, any other mammalian cells lacking the transgene will remain unaffected. Similarly, TK-NOG mice also support strong HBV replication property which were mainly used for drug screening purposes (52, 53). However, the demand for this mouse model is nowhere near as high when compared to the FRG KO strain due to male mice being infertile which ultimately result in poor breeding efficiency. More notably, a male wild-type NOG mouse is required to mate with a female TK-NOG mouse in order to successfully breed new transgenic pups followed by a very labor intense genotyping validation process.

## Dual Humanized Mouse Models

Although majority of these immunodeficient human liver chimeric mice have provided valuable insights on virology, the lack of a functional immune system impedes the study of human-specific immune responses triggered by HBV infection and immunotherapeutic strategies. Therefore, several groups including us have attempted to overcome these limitations by developing a dual humanized mouse model reconstituted with both hepatocytes as well as immune system of human origin (54). In fact, our group was one of the frontier labs in South East Asia to have previously demonstrated successful co-engraftment of human fetal liver-derived hematopoietic stem cells (HSCs) and hepatoblasts in an immunodeficient NOD-SCID IL-2rγ^−/−^ (NSG) mouse (HIL mouse) without any transgene modifications for the study of viral-related liver disease (55). These HIL mice were subjected to HCV inoculum before triggering HCV-specific immune responses which led to the development of liver pathogenesis like inflammation and fibrosis (56). Similarly, we have also utilized HIL mice to investigate the importance of intrahepatic CD206^+^ macrophages in HBV-induced liver inflammation and that liver fibrosis can be suppressed by anti-GM-CSF therapy (57, 58). While our HIL mice have indeed recapitulated most of the clinical symptoms observed in HBV/HCV patients, the weak liver chimerism gave rise to a much lower viral output when compared to some of the chimeric mice mentioned earlier. To overcome this obstacle, we have since utilized our own established immunodeficient NOD-SCID IL-2r^−/−^ (NIKO) mouse strain (59) to generate mice lacking the Fah gene herein, termed as Fah-NIKO mice. Like the FRG KO mice, Fah-NIKO mice also adapted a similar approach of utilizing NTBC cycling to facilitate mouse hepatocyte cell death allowing transplanted human hepatocytes to repopulate. Our preliminary data indicated that Fah-NIKO mice could also achieve high levels of human liver engraftment and support HBV infection for long periods (unpublished data). Although our NIKO mice have demonstrated good human hematopoietic reconstitution which is comparable to NSG mice (unpublished data), we have yet to examine the dual humanization capability of NIKO mice in response to HBV infection. Therefore, mice engrafted with both mature human immune system and humanized liver could hold the key to better assess human-specific immune responses triggered by HBV of which some of the recent developments of dual humanized mice will be highlighted below.

### AFC8-hu HSC/Hep Mice

In 2011, AFC8 mice became one of the first dual humanized mouse model to be established with human immune system and up to 30% repopulation of human hepatocytes in the mouse liver (60–62). Generation of these mice were actually quite similar to our HIL mice but with an added advantage of expressing a suicidal Caspase 8 inducible system to facilitate mouse hepatocyte cell death. Basically, immunodeficient Rag2/IL-2rγ KO mice in a BALB/c background were overexpressed with an albumin-driven Caspase 8 transgene which was fused with FK506 binding domain (FKBP) to specifically target mouse hepatocytes (AFC8 mice) (60). Following co-transplantation of human CD34^+^ HSCs and hepatocyte progenitor cells, these transgenic mice (AFC8-hu HSC/Hep mice) were administered with a FKBP dimerizer, AP20187 to induce hepatic injury for human liver engraftment. Similar to what we observed in HIL mice infected with HCV, AFC8-hu HSC/Hep mice also displayed human T cell responses to HCV and developed chronic liver inflammation/fibrosis which correlated with activation of stellate cells and human-specific fibrogenic genes (56, 60).

### A2/NSG/Fas-hu-HSC/Hep Mice

Since several reports indicated that chronic HBV-associated pathologies were related to infiltration of T lymphocytes and activated macrophages, the same group who generated AFC8-hu HSC/Hep mice developed another transgenic mouse model expressing HLA-A2 in an NSG background (A2/NSG) in order to study human antigen-specific T cell responses to HBV (63–65). They adapted a similar approach of transplanting HLA-A2 donor derived CD34^+^ HSCs and hepatic progenitors into A2/NSG pups but used a murine Fas activating antibody Jo2 to induce hepatotoxicity for engraftment of human hepatocytes (A2/NSG/Fas-hu-HSC/Hep mice) (66, 67). Mice that were infected with HBV displayed long-term viral persistence, robust expansion of human lymphoid T cells isolated from lymphoid and liver tissues following HBV antigen stimulation, and developed HBV-induced liver pathogenesis including hepatitis and fibrosis. More importantly, HBV-infected A2/NSG/Fas-hu-HSC/Hep mice exhibited high accumulation of activated human M2-like macrophages particularly at the fibrotic regions of the liver which was similarly observed in both patients with CHB and acute liver failure further demonstrating the importance of M2 macrophages in the innate immune system involving tissue remodeling and wound repair (63, 68). Indeed, the development of antigen-specific T cell responses have provided a unique advantage of utilizing these haplotype-matched dual humanized mice models for the study of HBV. However, it was believed that such immunosuppressive or pro-inflammatory phenotypes can be further optimized with improved engraftment of human hepatocytes in yielding higher viral titers return.

### uPA-NOG Transgenic Mice

As viral-induced liver pathogenesis including fibrosis, cirrhosis and even cancer can take decades to progress in humans, small animal models like dual humanized mice are ideal hosts to accelerate these processes for the study of its etiology. Hence, various groups have attempted to reconstitute functional human immune system in mice with high liver chimerism by tapping onto the uPA transgene technology and Fah KO strains (29, 54, 61, 69). For instance, the uPA transgene was expressed in a NOG mouse background instead of SCID to firstly stabilize its expression and expands the timeframe for a human cell transplantation (70). It was reported that the uPA expression in SCID mice would deteriorate with age which may affect the quality of liver humanization. Secondly, total body irradiation was replaced with treosulfan, a non-myeloablative conditioning method for engraftment of CD34^+^ HSCs. Although irradiation of mouse cells has been widely used prior to HSCs transplantation, treosulfan provided a safer and well-tolerated alternative to the more invasive method which may cause occasional mortality long term. Lastly, uPA-NOG transgenic mice can be reconstituted with mature human hepatocytes and HLA-mismatched HSCs from two separate donors. One common phenotypic feature shared in most humanized mouse models following transplants of fetal hepatoblasts was the low hepatic repopulation levels and failure of these epithelial cells to differentiate into its mature form fully. Subsequent methods like delivery of adenoviral vector-expressing human hepatocyte growth factor (HGF) into uPA-NOG mice was performed in hope of improving engraftment of fetal liver cells but this strategy proved to be unsuccessful (71, 72).

Although many have reported that 3–5% of human hepatocyte engraftment is sufficient to trigger virus-mediated intrahepatic immune responses and pathological changes, a much higher liver chimerism is required to further elucidate these characteristics (56–58, 60, 70). To overcome this challenge, adult human hepatocytes were transplanted into uPA-NOG mice resulting in >70% humanization of mouse liver together with functional human immune system derived from mismatched fetal liver HSCs. Since the supply of fetal liver tissues are becoming scarce due to enforcements of human biomedical research acts, the mismatched sample compatibility meant that HSCs can be obtained from alternative sources like cord blood banks. In fact, CD34^+^ HSCs successfully differentiated into specific immune cell subsets including CD3^+^ lymphocytes with a CD4:CD8 ratio similar to those established in an NSG background as well as mature human B cells in a donor-dependent manner (70, 73–75). Furthermore, the absence of haplotype restrictions between the two grafts provided more flexibility in generation of dual humanized mice without evidence of hepatocyte rejection by the human immune system. Although there were mild liver damages in some old mice, expansion of CD8^+^ T cells were absent and none of them developed signs of graft-vs.-host disease (GVHD) (70, 76). Hence, the successful engraftment of mismatched HSCs was clearly evidenced by low risk of cellular immune-mediated rejection of hepatocytes.

### FRGN Mice

Another very minute modification was made in FRG KO mice in order to harbor decent human hematopoietic engraftment as well as liver humanization. Although NSG mice remained one of the most conventional hosts for engraftment of HSCs, it has been demonstrated that immunodeficient mice in NOD background conferred a more superior support for human hematopoiesis due to the identification of SIRP-α polymorphism which primarily enhanced human CD47 ligand binding on mouse macrophages (77). Hence, FRG KO mice were intercrossed with NOD strains until all generations were homozygous for the four alleles herein, termed as FRGN mice (78). In fact, several advantages were observed in FRGN mice compared to FRG KO ones. Firstly, complete humanization of mouse liver was achieved quicker in the FRGN strain. Secondly, the average litter size was almost doubled in FRGN breeders and lastly, the average body weights for both mouse genders were significantly heavier (~5 g) than conventional FRG KO mice which may ultimately be critical for maintenance of a low mortality rate long-term. Like the use of treosulfan in uPA-NOG transgenic mice, FRGN mice were pre-conditioned with a DNA-damaging chemical, busulfan as well as ad-uPA prior to transplantation of mismatched CD34^+^ HSCs and adult hepatocytes intrasplenically (70, 78–80). NTBC cycling was performed accordingly to facilitate mouse hepatocyte cell death which in turn allow repopulation of human ones (43). Although both HSCs and hepatocytes were from two separate donors, FRGN mice displayed high hematopoietic reconstitution in blood, spleen, thymus, bone marrow, and liver organs along with high human hepatocyte replacement. More importantly, human blood, mature B cells, T cells, and Kupffer cells which plays a major role in pro-inflammatory responses were all detected in the liver of these mice (78). Although both uPA-NOG and FRGN mice demonstrated a marked improvement in dual reconstitution efficiency when utilizing mismatched HSCs and adult hepatocytes with minimal immune rejection, they were surprisingly not validated with some of the many potential applications such as HBV/HCV infection or metabolically induced steatohepatitis (70, 78).

### HIS-HUHEP Mice

It was only until more recently when another transgenic mouse strain was generated for the engraftment of both human immune system and mismatched adult hepatocytes. Basically, dual reconstitution efficiency was compared between BALB/c Rag2/IL-2rγ KO NOD.sirpa uPA transgenic mice transplanted with CD34^+^ fetal-derived HSCs alone (HIS), adult human hepatocytes alone (HUHEP), and both (HIS-HUHEP) (81). Similar to what was observed in the dual humanized mice models mentioned earlier, HIS-HUHEP mice hALB levels remained stable for long periods even in the presence of a supposedly allogeneic immune system, absolute numbers of blood leukocytes including CD3^+^ T cells retained its naïve phenotype without any immune expansion or activation and pro-inflammatory immune cell infiltration was absent in hepatocyte grafts suggesting that HIS-HUHEP mice could be the best candidate for investigating HBV-induced immune responses and developing liver pathogenesis *in vivo*. Hence, it was later demonstrated that HIS-HUHEP mice could indeed support chronic HBV infection displaying up to 10^9^ copies/ml viral DNA, both HBeAg and HBsAg measurements which was clinically equivalent in HBV^+^ patients and detectable HBV cccDNA (82). In addition, clusters of both CD3^+^ T cells and Kupffer cells were observed in HBV-infected HIS-HUHEP mice particularly around HBcAg^+^ human hepatocytes throughout the liver parenchyma. The robust increase of intrahepatic cytotoxic CD8^+^ T cells, activated NK cells and PD-1 mediated T cell exhaustion also indicated potential key effectors involved in an immunosuppressive environment (83). However, detection of antigen-specific T cell responses was absent due to the engraftment of HLA-mismatched grafts in HIS-HUHEP mice. Nevertheless, HBV-infected HIS-HUHEP mice treated with the nucleoside analog Entecavir (ETV) resulted in reduced HBV titers and restoration of naïve immune profiles evidenced by diminished liver immune cell infiltration suggesting that this dual humanized mouse model is suitable for potentially evaluating immunotherapeutic treatments (82, 84). One other aspect that remained to be investigated is HBV-mediated development of HCC. Although HBV-infected HIS-HUHEP mice could sustain high viremia and exhibited chronic inflammation phenotype, HCC development was not observed (82). Since HCC takes several decades to form, it may be difficult for dual humanized mice to recapitulate such HBV-associated liver pathology. Nevertheless, this *in vivo* platform could be helpful in elucidating tumorigenic pathways involving early phases of HCC initiation and progression.

### hBMSC-FRGS Mice

As accessibility to PHHs becomes more limited due to affordability or simply lack of healthy donors, several labs have started sourcing for *in vitro* alternatives. One prime example was the generation of human hepatocyte-like cells (hHLCs) derived from human induced pluripotent stem cells (hiPSCs) which required a three-step procedure of endoderm priming, hepatic specification and maturation (85–90). Although these hHLCs required very distinct culture conditions for differentiation, expansion and maintenance, these cells were well-differentiated and fully functional. In addition, engraftment of mature hHLCs was also successful in FRG KO-BALB/c SCID (FRGS) mice (hHLC-FRGS) displaying ~40% liver chimerism (87). More importantly, hHLCs and hHLC-FRGS mice were susceptible to chronic HBV infection completed with a full viral life cycle which was efficiently blocked by MyrB and ETV. The same research group then adapted a similar approach in exploring the possibility of generating a dual humanized mouse model by using human bone mesenchymal stem cells (hBMSCs) (91). Basically, hBMSCs were isolated from bone marrows of healthy male volunteers and cultured in multilineage (osteocytes, adipocytes and HLCs) differentiation media prior to transplantation into FRGS mice (hBMSC-FRGS). Unlike hHLC-FRGS mice, hBMSC-FRGS actually displayed higher liver chimerism (58.7%) including HLA^+^ cells that were also positive for mature human hepatocyte-specific markers. Furthermore, varying amounts of hCD45^+^ cells were detected in bone marrows, thymus, lymph node, spleen, liver, and peripheral blood of hBMSC-FRGS mice. More notably, multiple human immune cell lineages such as T cells, B cells, NK cells, macrophages, and dendritic cells were present in the mouse liver following transplantation of hBMSCs. Similar to HIS-HUHEP mice as mentioned earlier, hBMSC-FRGS mice support persistence HBV infection with high levels of HBV DNA, HBsAg, HBeAg, as well as detectable intrahepatic HBV cccDNA (82, 91). Large production of human-derived pro-inflammatory cytokines/chemokines triggered by specific immune cell subsets was also released and sustained throughout the course of infection which may contribute to liver immunopathological injury. Critically, HBV-infected hBMSC-FRGS mice developed acute/chronic hepatitis patterns with varying degrees of lymphocytic portal inflammation, liver fibrosis, accumulations of scar tissues and ultimately progressed to liver cirrhosis which was similarly observed in CHB patients. Thus, this dual humanized mouse system could possibly be the most ideal model for evaluating viral immune pathophysiology and refining antiviral therapeutics.

## Conclusion

Many research groups have deciphered some of the basic concepts of virus-host interactions by utilizing conventional platforms like *in vitro* culture systems as well as wild-type/transgenic mice which have been instrumental in the evolution of humanized mouse models. The generation of human liver chimeric mice was the first model to permit long term HBV persistence which were mainly used for understanding HBV life cycle and identification of potential antiviral drug targets. Over time, improvements led to the development of dual humanized mice engrafted with high liver chimerism and human immune cell lineages to better investigate HBV-triggered human immune responses. Concurrently, these HBV-infected mice developed severe pathological changes including chronic inflammation and fibrosis/cirrhosis further recapitulating liver pathogenesis observed in CHB patients. However, development of HCC *in vivo* remains elusive which is high likely due to its unpredictable proliferative nature to form over decades in humans. Although usage of dual humanized mice has yielded much progress in the field of HBV research as highlighted in this review (summarized in Table 1), improved models are required to incorporate the missing transition link of chronic HBV and HCC in hope of moving one step closer toward HBV cure.

**Table 1:** Summary of humanized mouse models for the study of HBV.

**Types**	**Advantages**	**Limitations**	**References**
HBV-Trimera	• First immunodeficient mouse model transplanted with *ex vivo* human liver tissues isolated from HBV^+^ patients • Hepatect and reverse transcriptase inhibitors reduced viremia *in vivo*	• Low viremia • Absence of HBV persistence • Very short lived functional human hepatocytes	(31)
uPA-SCID	• First transgenic mouse model to repopulate human hepatocytes in diseased livers of mouse recipients • Exhibited high levels of HBV replication • Good pre-clinical model for anti-viral applications	• Constitutive liver toxicity resulted in high mortality • Poor breeding capacity • Unpredictable mouse colonies	(32–42)
FRG KO	• Mouse hepatic injury can be controlled with NTBC cycling • Immunodeficient background to permit better human hepatocyte engraftments • Robust expansion of mature human hepatocytes (mg/ml hALB levels) • Exhibited high levels of viremia and persistent HBV • Suitable for studying HBV life cycle • Good pre-clinical model for anti-viral applications	• Absence of HBV-induced human immune responses • Absence of liver pathogenesis	(43, 50)
TK-NOG	• Immunodeficient background which requires non-toxic drug GCV to destroy mouse hepatocytes in order to accommodate human ones • Exhibited high levels of viremia • Mainly used for drug screening purposes	• Poor breeding capacity • Male mice are infertile. Requires genotyping for pups • Absence of HBV-induced human immune responses • Absence of liver pathogenesis	(51–53)
HIL	• Human immune liver mice generated by transplantation of CD34^+^ HSCs derived from fetal liver • Exhibited functional human T cell responses toward HCV/HBV and developed liver pathogenesis • Identification of anti-GM-CSF therapy against HBV-induced liver fibrosis	• Very low human hepatocyte reconstitution • Low viremia	(56–58)
AFC8	• First dual humanized mouse model established with functional human immune system and up to 30% humanized mouse liver • Generated by co-transplantation of CD34^+^ HSCs and hepatic progenitors from same donor • Exhibited functional human T cell responses toward HCV and developed liver inflammation	• Hepatic progenitors did not fully differentiate into mature human hepatocytes • Yet to be demonstrated in a HBV infection setting	(60, 62)
A2/NSG/Fas	• Transgenic mouse model expressing HLA-A2 to study antigen-specific T cell responses to HBV • Generated by co-transplantation of CD34^+^ HSCs and hepatic progenitors from same donor • Murine Jo2 induced mouse liver damage • Mice displayed long term viral persistence • Mice developed HBV-induced liver fibrosis, infiltration of T lymphocytes and high accumulation of macrophages	• Hepatic progenitors did not fully differentiate into mature human hepatocytes • Persistent HBV but with low viral titers	(63–65)
uPA-NOG	• uPA expression on NOG mouse strain has longer lifespan compared to SCID background • Irradiation was replaced with treosulfan for HSC engraftment • Generated with HLA-mismatched HSCs and mature human hepatocytes from different donors • uPA-NOG mice displayed hemato-lymphoid reconstitution and expansion of human hepatocytes • No signs of GVHD	• Yet to be demonstrated in a viral infection setting	(70)
FRGN	• Quicker complete liver humanization than FRG KO mice • Larger litter size & body weight compared to FRG KO mice • Irradiation was replaced with busulfan • Generated with HLA-mismatched HSCs and mature human hepatocytes from different donors • Displayed hemato-lymphoid reconstitution and expansion of human hepatocytes • No signs of GVHD	• Yet to be demonstrated in a viral infection setting	(78)
HIS-HUHEP	• Generated with HLA-mismatched HSCs and mature human hepatocytes from different donors • Displayed hemato-lymphoid reconstitution and expansion of human hepatocytes • No signs of GVHD • Exhibited high levels of viremia and persistent HBV • Nucleoside analogs reduced viral titers and restored naïve human immune profiles	• Detection of antigen-specific T cell responses was absent • Liver fibrosis and development of HCC was absent	(81–84)
FRGS	• Human hepatocyte-like cells (hHLCs) derived from hIPSCs can differentiate and expand in FRGS mice (~40% liver chimerism) • Human bone mesenchymal stem cells (hBMSCs) can differentiate and expand in FRGS mice with ~58.7% liver chimerism in addition to multiple human immune cell lineages • Exhibited high levels of viremia and persistent HBV • HBV-infected hBMSC-FRGS mice developed chronic inflammation, liver fibrosis and cirrhosis	• Development of HCC was absent	(87, 91)

## Author Contributions

FL took the lead in writing the manuscript. CW and QC contributed to writing. QC supervised the preparation of manuscript. All authors studied the literature and approved the submitted version.

## Conflict of Interest

The authors declare that the research was conducted in the absence of any commercial or financial relationships that could be construed as a potential conflict of interest.
